# Characterization and Gel Properties of Low-Molecular-Weight Carrageenans Prepared by Photocatalytic Degradation

**DOI:** 10.3390/polym15030602

**Published:** 2023-01-24

**Authors:** Chen Song, Ying You, Chengrong Wen, Yinghuan Fu, Jingfeng Yang, Jun Zhao, Shuang Song

**Affiliations:** 1 National Engineering Research Center of Seafood, School of Food Science and Technology, Dalian Polytechnic University, Dalian 116034, China; 2 National & Local Joint Engineering Laboratory for Marine Bioactive Polysaccharide Development and Application, Dalian Polytechnic University, Dalian 116034, China; 3 College of Food Science and Engineering, Jilin Agricultural University, Changchun 130118, China; 4 School of Light Industry and Chemical Engineering, Dalian Polytechnic University, Dalian 116034, China

**Keywords:** degraded carrageenan, photocatalysis degradation, polysaccharide degradation, carrageenan oligosaccharides, photocatalyst TiO_2_

## Abstract

Low-molecular-weight carrageenan has attracted great interest because it shows advantages in solubility, absorption efficiency, and bioavailability compared to original carrageenan. However more environment-friendly and efficient methods to prepare low-molecular-weight carrageenan are still in great need. In the present study, a photocatalytic degradation method with only TiO_2_ has been developed and it could decrease the average molecular weight of κ-carrageenan to 4 kDa within 6 h. The comparison of the chemical compositions of the degradation products with those of carrageenan by FT-IR, NMR, etc., indicates no obvious removement of sulfate group, which is essential for bioactivities. Then 20 carrageenan oligosaccharides in the degradation products were identified by HPLC-MS^n^, and 75% of them possessed AnGal or its decarbonylated derivative at their reducing end, indicating that photocatalysis is preferential to break the glycosidic bond of AnGal. Moreover, the analysis results rheology and Cryo-SEM demonstrated that the gel property decreased gradually. Therefore, the present study demonstrated that the photocatalytic method with TiO_2_ as the only catalyst has the potential to prepare low-molecular-weight carrageenan with high sulfation degree and low viscosity, and it also proposed the degradation rules after characterizing the degradation products. Thus, the present study provides an effective green method for the degradation of carrageenan.

## 1. Introduction

Carrageenans, a family of highly sulfated polysaccharides, are derived from marine red algae, such as *Eucheuma*, *Gigartina*, *Chondrus*, and *Hypnea* [1]. According to the number and position of the sulfate groups, they can be classified into kappa (κ)-, iota (ι)-, and lambda (λ)–carrageenan. Among them, κ-carrageenan, which is mainly composed of D-galactose-4-sulfate (G4S) and 3,6-anhydro-D-galactose (An), demonstrates the lowest viscosity, and it is universally used as thickeners, gelling agents, and stabilizers in processing foods, including yogurt, ice cream, and some processed meats [2].

Recently, various biological activities of κ-carrageenan such as antiviral, antioxidant, anticoagulant, and anti-obesity effects have attracted widespread attention [3,4,5,6]. However, κ-carrageenan has a large molecular weight, poor solubility, and low bioavailability, which greatly limit its application range [7]. As the degradation products of carrageenan, carrageenan oligosaccharides exhibited diverse biological activities as well as other remarkable properties, especially better solubility and higher absorption [8]. In addition, carrageenan oligosaccharides also have potential as an antimicrobial agent [9], antifreeze agent [10], and drug delivery carrier [11].

Therefore, it is meaningful to develop the degradation methods to prepare low-molecular-weight carrageenan with excellent solubility so as to improve its biological activities and expand its application range. The reported degradation methods for κ-carrageenan include acid, enzymatic, microwave-assisted, ultrasonic-assisted, and oxidative degradation [12,13,14]. The operation process of acid degradation polysaccharide is relatively simple and costs less, but it is difficult to master the conditions of the acid degradation polysaccharide process, and easy to damage the structure of the polysaccharide, such as the sulfate groups, thus affecting its biological activities [15]. The enzymatic approach is high-cost and inefficient, which has limited its industrial-scale applications. Although the microwave-assisted and ultrasonic-assisted degradation are simple and non-pollution, their production costs are also high due to their necessary equipment [16]. Therefore, it is essential to develop a high-efficiency, environmentally friendly, and low-cost degradation method, which can retain the sulfate groups of κ-carrageenan.

**Figure 1:**
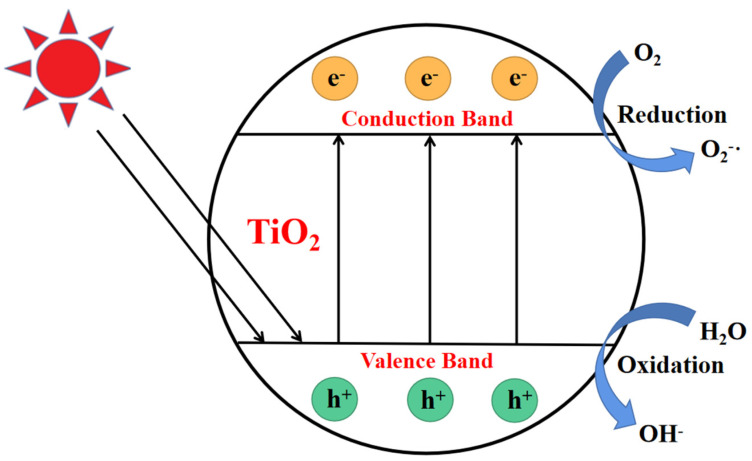
Photocatalytic formation mechanism of electron−hole pair in a semiconductor TiO_2_ particle.

Photocatalytic degradation, as an advanced oxidation process, has made rapid progress since the discovery of the first water splitting reaction using a semiconductor in 1972 by Fujishima and Honda. As shown in Figure 1, the photocatalytic process begins from the semiconductor band gap. When an electron (e^−^) in a semiconductor valence band is excited to transition to the conduction band, photogenerated holes (h^+^) in the valence band will be generated in response, thus yielding electron-hole (e^−^ and h^+^) pairs inside the semiconductor [17]. As a result, the excited electrons will produce free radicals to impel the photocatalytic reactions. Among various semiconductor catalysts, titanium dioxide (TiO_2_) is one of the best photocatalysts for inducing a series of reductive and oxidative reactions on its surface, as TiO_2_ is the most active photocatalyst under the photon energy of 300 nm < λ < 390 nm, and it remains stable after several catalytic cycles [18]. Besides this, TiO_2_ catalyst also has many functional properties, such as its strongly thermal stability and resistance to chemical damage, which promote its wide application in photocatalytic technology [19].

Previous research revealed that photocatalysis showed a good performance in the treatment of organic pollutants due to its advantages of fast reaction speed, low cost, and “green chemistry” technology [20]. At present, photocatalysis technology is applied in not only the removement of organic pollutants, but also anti-virus, anti-bacteria, etc. [21,22]. Photocatalytic degradation shows the advantages of high-efficiency, energy-saving, ecofriendly, and sustainability [23]. More importantly, it is more suitable for sulfated polysaccharide degradation because it does not obviously remove the sulfate group, which is critical for the bioactivities [24]. Photocatalysis with the addition of H_2_O_2_ could degrade chondroitin sulfate [25] and fucoidan [26]. However, there is no report on the successful photocatalysis degradation of sulfated polysaccharides without H_2_O_2_ to date.

In the present study, a photocatalytic method with only TiO_2_ as photocatalyst was developed for κ-carrageenan degradation, and the resulting degradation products were detected by a series of analytical methods to reveal changes of κ-carrageenan in the structure, composition, rheological property, and microstructure during photocatalytic degradation.

## 2. Materials and Methods

### 2.1. Chemicals

κ-carrageenan was obtained from Aladdin Co. Ltd. (Shanghai, China). Titanium dioxide (TiO_2_) nanoparticles (25 nm) were obtained from Evonik Degussa Specialty Chemicals Co., Ltd. (Frankfurt, Germany). All dextran standards were provided by Sigma-Aldrich (St. Louis, MO, USA). Other reagents were purchased from Sinopharm Chemical Reagent Co., Ltd. (Shanghai, China).

### 2.2. Photocatalytic Degradation of κ-Carrageenan

A CEL-HXF300-T3 system (Zhongjiao Jinyuan Technology Co., Ltd., Beijing, China) was used for the photocatalytic reaction, and it was configured with a 300 W Xenon lamp whose wavelength range is 300 nm–2500 nm (PerkinElmer, Waltham, MA, USA). 

κ-carrageenan (1 g) was dissolved in water (200 mL), magnetic stirring at 60 °C for 6 h, and placed in the refrigerator at 4 °C overnight to obtain κ-carrageenan solution (0.5% *w*/*w*). The κ-carrageenan solution was added into the photocatalytic reactor, and TiO_2_ (5%), the combination of H_2_O_2_ (300 mM) and TiO_2_ (5%), or H_2_O_2_ (300 mM) were added while stirring (the addition amounts of TiO_2_ and H_2_O_2_ were based on the results optimized by our team previously [26]). During the photocatalytic experiment, the solution was continuously stirred and exposed to a Xenon lamp for 6 h, and the samples degraded for 2 h, 4 h, and 6 h. After centrifugation at 10,000 rpm for 20 min, the supernatants were lyophilized individually, which were named as DC-2, DC-4, and DC-6, respectively.

### 2.3. Chemical Composition Analysis

The total sugar content was measured by phenol–sulfuric acid method using galactose as the standard [27]. The reducing sugar content was measured by 3,5-dinitrosalicylic acid (DNS) assay using galactose as the standard [28]. The uronic acid content was determined by m-hydroxy biphenyl method using D-galacturonic acid as the standard [29]. The sulfated polysaccharide content was determined by the metachromatic assay with 1,9-dimethylmethylene blue (DMB) using pure κ-carrageenan as the standard [30]. The 3,6-anhydro galactose content was measured by resorcinol method with a minor modification [31]. The total sulfate group content was determined by the barium chloride-gelatine method using anhydrous potassium sulfate as the standard [32]. The free sulfate content was determined through ion chromatography using a DIONEX IonPac AS23 column (4.0 mm × 250 mm) at 30 °C and 4.5 mM Na_2_CO_3_ and 0.8 mM NaHCO_3_ were utilized as mobile phases.

(1) The sulfate content = Total sulfate content−Free sulfate content

### 2.4. Molecular Weight Determination

The molecular weights (Mw) of samples were examined by high performance gel permeation chromatography (HPGPC), which was configured with a refractive index detector (Waters 2414, Milford, MA, USA) and a TSK G5000PWXL chromatographic column (7.8 mm × 300 mm) was used for separation. The sample was eluted with 100 mM ammonium acetate at a flow rate of 0.4 mL/min. A series of dextran standards with molecular weights of 5, 25, 50, 410, and 670 kDa were applied as standards.

### 2.5. Thin Layer Chromatography (TLC)

Each 2 μL of polysaccharide samples and standards (glucose, lactose, and β-cyclodextrin) were spotted separately onto a 20 cm × 20 cm plastic TLC plate (Macherey, Düren, Germany), and the plate was performed with the solvent system of n-butanol: formic acid: water (2:1:1, *v*/*v*/*v*) at 25 °C. Then, the plate was stained with orcinol–sulfuric acid reagent which consisted of 50 mg of orcinol and methanol: H_2_SO_4_ (5:1, *v*/*v*), and then heated at 120 °C to visualize the bands.

### 2.6. Fourier-Transform Infrared (FT-IR) Spectroscopic Analysis

The carrageenan and its degradation products (2 mg) were individually pressed into 1 mm pellets with KBr (100 mg). The FTIR spectra of sample pellets were recorded in a dry atmosphere at room temperature (21 ± 1 °C) by using a Spectrum One-B FTIR Spectrometer (PerkinElmer, Waltham, MA, USA) in the range of 500 to 4000 cm^− 1^. Furthermore, a background spectrum was subtracted automatically by using pure dried KBr to eliminate the interferences of carbon dioxide and water in the atmosphere.

### 2.7. Nuclear Magnetic Resonance (NMR) Analysis

DC-6 (50 mg) in 500 μL of D_2_O (99.9%) was dissolved and lyophilized three times. ^1^H NMR, ^13^C NMR, and 2-dimensional heteronuclear single quantum correlation (HSQC) of DC-6 were recorded on a Bruker Avance III 400 NMR spectrometer (Bruker, Rheinstetten, Germany).

### 2.8. Mass Spectrometry Analysis

DC-6 was further fractionated with a molecular weight cut-off (MWCO) of 3 kDa to obtain κ-carrageenan oligosaccharides, which were further subjected to mass spectrometry analysis according to our previously reported method with minor modification [33]. Briefly, the oligosaccharides were labeled with 1-phenyl-3-methyl-5-pyrazolone (PMP) at first, and then the products were analyzed by HPLC-MS^n^. LXQ mass spectrometer (Thermo Fisher, Pittsburgh, PA, USA) coupled with a photodiode array detector (PDA) and an electrospray ionization (ESI) source were employed. The oligosaccharide derivatives were separated with a TSKgel-Amide-80 column (4.6 mm × 150 mm, 3 μm) and eluted with 20 mM ammonium acetate-acetonitrile (5:95, *v*/*v*) at 0.2 mL/min. For the electrospray ion source, the spray voltage was set at 4.5 kV, the capillary voltage was set at 37 V, and the capillary temperature was 275 °C [34]. All data were acquired and processed through XCalibur software.

### 2.9. Rheological Measurement

The dynamic rheological measurement of the samples was obtained with a DHR-2 rheometer (TA Instruments, New Castle, DE, USA). A 60-mm diameter aluminum plate geometry was used for measurement at the distance of 500 μm. First of all, about 2 mL of the sample was taken and placed on the sample plate, the excess sample was scraped off, coated with silicone oil, and covered with a moisturizing cap to minimize measurement errors. Before the test, the initial temperature equilibrium of the samples was maintained for 5 min. A strain sweep program (0.001–100% at 25 °C and 1 Hz) was performed to determine the linear viscoelastic region. The time sweeps were conducted in the scope of 0–160 s at 4 °C and 25 °C, and the frequency sweeps were carried out in the range of 0.01–10% at 4 °C and 25 °C.

### 2.10. Cryogenic Scanning Electron Microscopy (Cryo-SEM)

The microstructure of the samples was carried out by using a Cryo-SEM (SU8010, Hitachi, Japan) configured with the PP3010T preparation system (Quorum Technologies Ltd., East Sussex, UK). Briefly, an appropriate amount of sample was loaded onto rivet and frozen quickly in liquid nitrogen slush. After that, the fractured samples were further sublimated at −60 °C for 30 min and coated with gold for 30 s. At last, the samples were transmitted to the SEM chamber for observation [35].

### 2.11. Statistical Analysis

All the results were presented as mean ± SD. The statistical analysis was performed using one-way ANOVA multiple-comparisons tests by SPSS 19.0 statistical software. *p* < 0.05 was considered to be statistically significant between the groups.

## 3. Results and Discussions

### 3.1. Photocatalytic Degradation of Carrageenan

**Figure 2:**
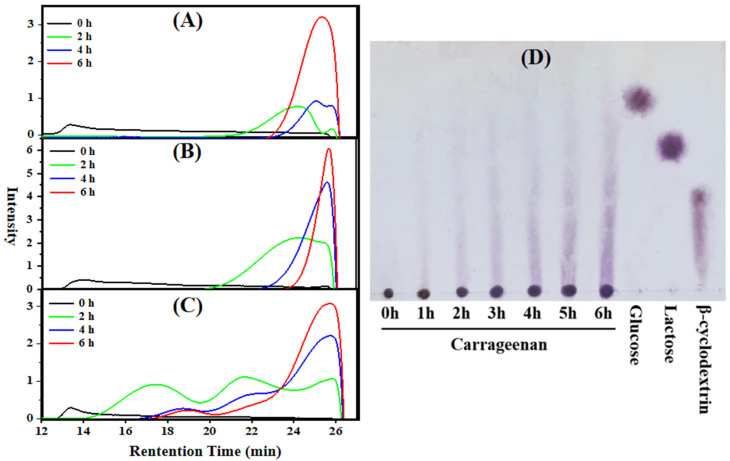
Molecular weight distribution changes of carrageenan during photocatalytic reaction monitored by HPGPC. (**A**) 300 mM H_2_O_2_, (**B**) 5% TiO_2_ and 300 mM H_2_O_2_, (**C**) 5% TiO_2_, and (**D**) TLC with 5% TiO_2_ of degraded carrageenan.

In order to reveal the molecular weight change of κ-carrageenan in the photocatalytic reaction, HPGPC and TLC were used to analyze the hydrolysates during the photocatalytic reaction. We compared the degradation effects of 300 mM H_2_O_2_ (Figure 2A), the combination of 300 mM H_2_O_2_ and 5% TiO_2_ (Figure 2B), and 5% TiO_2_ (Figure 2C) on carrageenan. Comparison of the molecular distribution of products collected at 2 h and 4 h indicates that the combination of H_2_O_2_ and TiO_2_ possesses the highest degradation efficiency, followed by using H_2_O_2_ alone, then TiO_2_. However, all of the three conditions could degrade carrageenan to 3~6 kDa after 6 h. Notably, it is unexpected that a similar outcome of 6 h degradation could also be achieved by using only TiO_2_. TLC analysis (Figure 2D) also demonstrated that oligosaccharides gradually accumulated by using TiO_2_ alone.

TiO_2_ as photocatalyst could generate electron-hole (e^−^ and h^+^) pairs under the light, and the excited electrons can further generate free radicals. H_2_O_2_, which could also produce radicals, is widely applied in the degradation of polysaccharides [36]. We have reported the photocatalytic degradation of fucoidan and chondroitin sulfate with TiO_2_ as well as H_2_O_2_, and the products of 3~5 kDa were obtained after a 6 h reaction [25,26]. As a strong oxidizing agent, H_2_O_2_ could promote the oxidation degradation [37], but it is harmful to health and unfriendly to environment [38]. Moreover, its addition could increase the production cost.

In the present study, 4 kDa degradation product of κ-carrageenan was produced through a 6 h reaction without H_2_O_2_. Of note, fucoidan and chondroitin sulfate could not be degraded obviously within 6 h (data not published). The efficient degradation of κ-carrageenan by photocatalytic reaction without H_2_O_2_ may be attributed to its structure. Notably, the results showed that sulfated polysaccharides can be effectively degraded by photocatalytic degradation method with only TiO_2_, which is a significant discovery.

### 3.2. Structure Changes of Carrageenan by Photocatalytic Degradation

**Figure 3:**
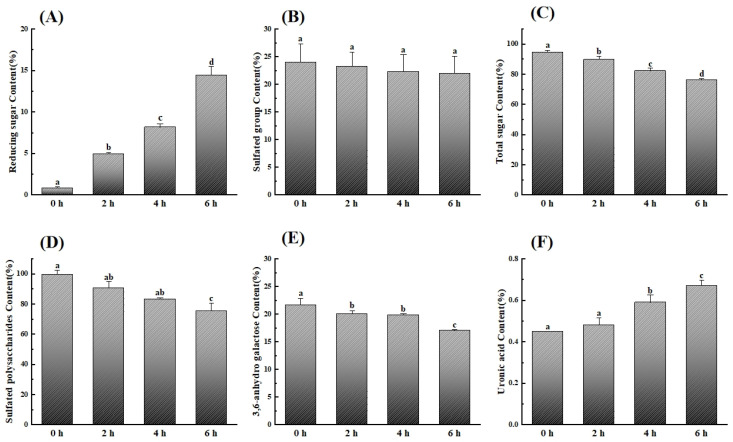
Changes in chemical compositions of κ-carrageenan. (**A**) Reducing sugar content, (**B**) sulfated group content, (**C**) total sugar content, (**D**) sulfated polysaccharide content, (**E**) 3,6-anhydro galactose content, and (**F**) uronic acid content. Data are expressed as the mean ± SD (n = 3). Graph bars marked with different letters on top represent statistically significant results (*p* < 0.05) based on one-way analysis of variance (ANOVA) with Duncan’s range tests, whereas bars marked with same letter correspond to results that show no statistically significant differences.

In order to monitor the chemical structure changes of κ-carrageenan during the photocatalytic reaction, colourimetry, FT-IR spectroscopy, and NMR spectroscopy were applied. With the extension of photocatalytic time, the reducing sugar content increased from 0.85% to 14.41% (Figure 3A), indicating the effective degradation by photocatalysis, which was consistent with molecular weight change. At the same time, no significant difference in sulfate group content was observed between κ-carrageenan and its degradation products (Figure 3B). However, the total sugar content, sulfated polysaccharide content, and 3,6-anhydro galactose content all decreased by around 20% after 6 h degradation (Figure 3C–E), which reflected the breakdown proportion during the degradation. Of note, the uronic acid content showed an increasing trend, suggesting the byproduct as uronic acid (Figure 3F).

**Figure 4:**
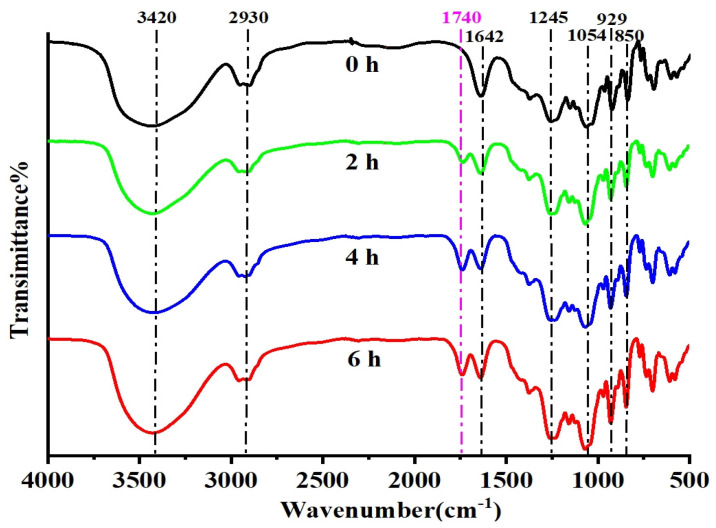
FT−IR spectra of κ-carrageenan after 0 h, 2 h, 4 h, and 6 h photocatalytic reaction.

The FT-IR spectra of κ-carrageenan, DC-2, DC-4, and DC-6 were presented in Figure 4. The absorption band at 1642 cm^−1^ was attributed to the stretching vibration of C=O group [39]. The presence of signals at 1245 cm^−1^ and 850 cm^−1^ were assigned, respectively, to O=S=O symmetric vibration and to –O–SO_3_ stretching vibration at C-4 of the galactose ring. Importantly, the peak position, shape, and intensity of the presence of signals at 850 cm^−1^ showed that sulfate group was retained during the photocatalytic reaction. The absorption band at 929 cm^−1^ was attributed to the C–O–C vibration of the 3,6-anhydro-D-galactose residue [40]. Furthermore, the absorption band at 1054 cm^−1^ was attributed to the stretching vibration of C-O-C on the sugar ring, and this peak was the characteristic absorption peak of pyranose ring. Notably, although great similarities were observed between κ-carrageenan and its degradation products, an absorption band at 1740 cm^−1^ corresponding to the stretching vibration of C=O grew with the extension of photocatalysis degradation, indicating the formation of carboxyl or aldehyde group [41,42].

**Figure 5:**
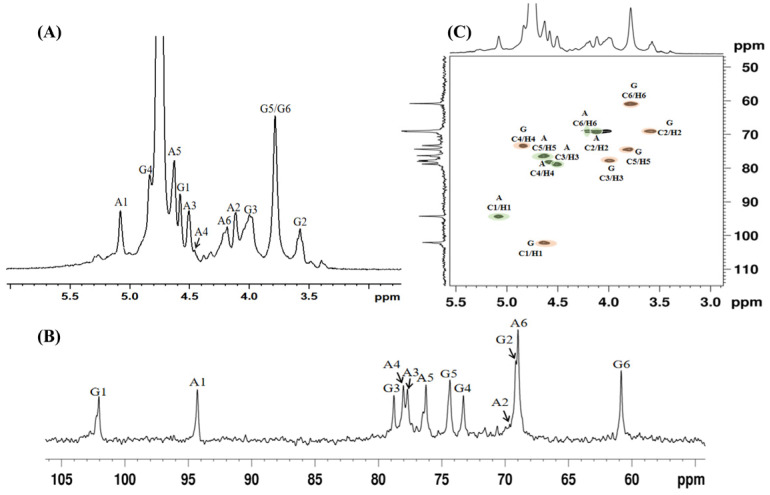
(**A**) ^1^H NMR, (**B**) ^13^C NMR, and (**C**) HSQC spectra of DC-6. Correlation peaks are marked on the spectrum: G4S (G)—orange and AnGal (A)—green.

To further explore the possible structural changes caused by photocatalysis degradation, ^1^H NMR, ^13^C NMR, and HSQC spectra (Figure 5) of DC-6 were investigated. The cross peaks at 4.63/102.1 ppm and 5.08/94.5 ppm in the HSQC spectrum were identified as anomeric proton/carbon signals of Gal4S and AnGal, respectively. Other ^1^H and ^13^C chemical shifts were also assigned in Table 1. Comparison of the NMR signals with those of κ-carrageenan [43,44] indicates that DC-6 has no obviously structural changes. It is worth mentioning that no carbon signal was observed at 66.4 ppm corresponding to the C-4 position of galactose without sulfate substitutions [45], indicating no obvious desulphurization occurs in the photocatalytic degradation.

### 3.3. Identification of Carrageenan Oligosaccharides Produced by Photocatalytic Degradation

**Figure 6:**
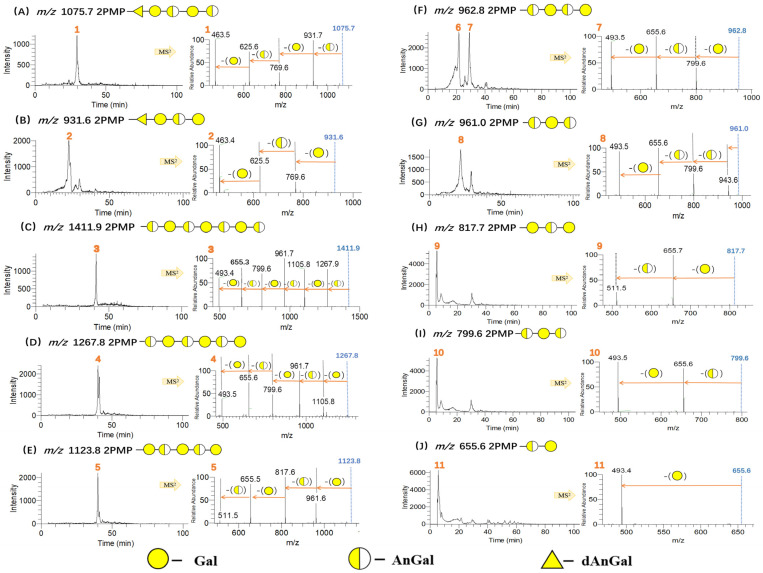
HPLC-MS^n^ analysis results of carrageenan oligosaccharides in DC-6. The identified peaks are numbered.

The oligosaccharides in DC-6 were characterized by ESI-MS^n^ in positive ion mode after PMP derivatization, and their extracted ion chromatograms and MS^2^ spectra were shown in Figure 6. The oligosaccharides were identified mainly based on their pseudo-molecular ion and product ions. As illustrated in Figure 6A, the pseudo-molecular ion of Peak 1 at *m*/*z* 1075 [M+H]^+^ gave product ions at *m*/*z* 931 [M-AnGal+H]^+^, *m*/*z* 769 [M-AnGal-Gal+H]^+^, *m*/*z* 625 [M-AnGal-Gal-AnGal+H]^+^, and *m*/*z* 463 [M-AnGal-Gal-AnGal-Gal+H]^+^. Thus, Peak 1 was considered to be a PMP-labeled pentasaccharide composed of (Gal)_2_, (AnGal)_2_ and a residue of 114 Da (named as dAnGal). Then, the oligosaccharide of Peak 1 was elucidated as AnGal→Gal→AnGal→Gal→dAnGal. Some fragments of Peak 1 were also observed in the MS^2^ of Peak 2 (Figure 6B), including *m*/*z* 769 [M-AnGal-Gal+H]^+^, *m*/*z* 625 [M-AnGal-Gal-AnGal+H]^+^, and *m*/*z* 463 [M-AnGal-Gal-AnGal-Gal+H]^+^. Therefore, Peak 2 was inferred as a derivative of Peak 1 losing an AnGal, so it was identified as Gal→AnGal→Gal→dAnGal.

As presented in Figure 6C, the pseudo-molecular ion of Peak 3 was at *m*/*z* 1411 [M+H]^+^, which gave product ions at *m*/*z* 1267 [M-AnGal+H]^+^, *m*/*z* 1105 [M-AnGal-Gal+H]^+^, *m*/*z* 961 [M-AnGal-Gal-AnGal+H]^+^, *m*/*z* 799 [M-AnGal-Gal-AnGal-Gal+H]^+^, *m*/*z* 655 [M-AnGal-Gal-AnGal-Gal-AnGal+H]^+^, and *m*/*z* 493 [M-AnGal-Gal-AnGal-Gal-AnGal-Gal+H]^+^ in MS^2^. Thus, Peak 3 was speculated as a PMP-labeled heptasaccharide, AnGal→Gal→AnGal→Gal→AnGal→Gal→AnGal. Similarly, oligosaccharides from disaccharide to hexasaccharide constructed with the alternate AnGal and Gal residues were found according to Figure 6D–J.

In the present study, a total of 20 oligosaccharides in the photocatalytic degradation products were identified, including di-, tri-, tetra-, penta-, hexa-, and hepta-saccharides (Table 2), and their relative yields could be reflected by their chromatogram peak areas. It is worth noting that sulfate content in the identified oligosaccharides is not as high as it has been determined in Section 3.2. This is due to the fragmentation through loss of sulfate groups and sensitivity problems of mass spectroscopy detection, particularly when analyzing oligosaccharides substituted with many sulfate groups [46,47].

**Figure 7:**
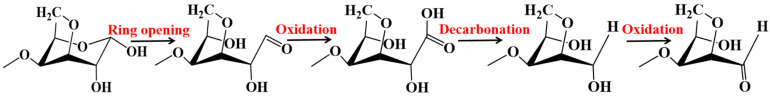
Proposed chemical structure change of AnGal during photocatalytic reaction.

The possible formation process of dAnGal was proposed in Figure 7. After the break of glycosidic bond of AnGal, the hexatomic ring of AnGal is opened. Then the new reducing end is subjected to a rapid oxidation to form the carboxyl group. Next, the decarbonation and oxidation reactions occur subsequently to generate dAnGal.

**Figure 8:**
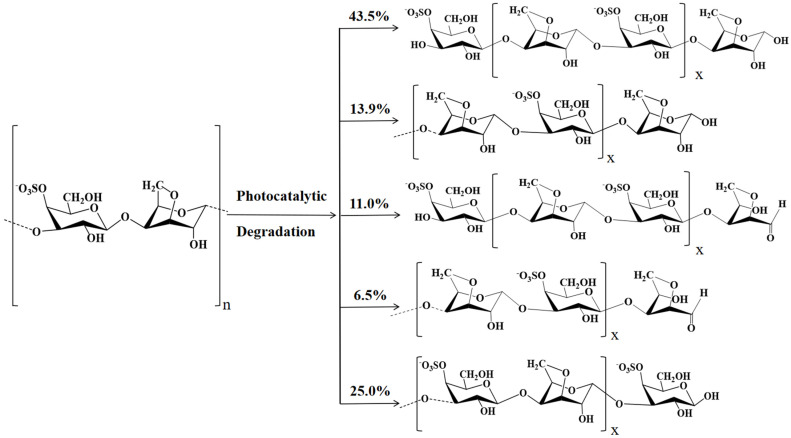
Carrageenan oligosaccharides produced during photocatalytic reaction (x = 0, 1, 2, 3…).

Moreover, the photocatalytic depolymerization rule was proposed in Figure 8 according to the degradation products. The major proportion (57.4%) of the degradation products demonstrated AnGal at their reducing end, and 17.5% was its decarbonylated derivative, dAnGal, at the reducing end. This suggests that AnGal’s glycosidic bond is more ready to be attacked by free radicals. Notably, the rule of photocatalytic depolymerization was different from those of other depolymerization methods. Carrageenase could degrade κ-carrageenan to yield even-numbered oligosaccharides with AnGal at the non-reducing end and Gal at the reducing end [48], while acid hydrolysis could produce odd-numbered oligosaccharides with Gal at both the reducing and non-reducing ends [49]. In the present study, similar amounts of odd and even numbers of κ-carrageenan oligosaccharides were obtained by photocatalytic degradation, and more AnGal or its derivative were observed at the reducing ends. Thus, the degradation result of photocatalytic reaction was different from those of enzymatic degradation and acid hydrolysis.

### 3.4. Rheological Properties of Carrageenan Degradation Products

**Figure 9:**
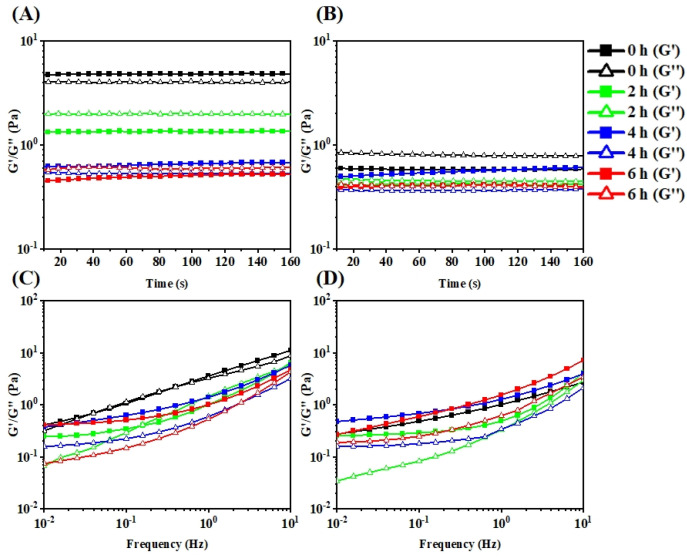
The storage modulus (G′) (solid symbols), loss modulus (G″) (open symbols) of κ-carrageenan, DC-2, DC-4, and DC-6 under the time range of 0–160 s at (**A**) 4 °C and (**B**) 25 °C and under the frequency scope of 0.01–10% at (**C**) 4 °C and (**D**) 25 °C.

In order to monitor the predominant elasticity and gel-like behavior of carrageenan during the photocatalytic degradation, the elastic (G′) and viscous (G″) moduli of carrageenan, DC-2, DC-4, and DC-6 in the time range of 0–160 s and the frequency sweep of 0.1–10 Hz were depicted. As shown in Figure 9, κ-carrageenan at 0.5% (*w*/*w*) could form a weak gel at 4 °C, and did not show obvious gel properties at 25 °C. At the same time, its degradation products, including DC-2, DC-4, and DC-6 all failed in the gel formation at both 4 °C and 25 °C. Moreover, their performance had not been improved with the change of frequency. It has been revealed that 3,6-anhydro-D-galactose residues allow a helicoidal secondary structure, which plays a crucial role in the formation of carrageenan gels [50]. Although the photocatalytic reaction did not break the structure blocks of carrageenan obviously, its gel property was deprived with the decrease of molecular weight. This is due to the length and the degree of entanglement of the molecular chain, which will be decreased during the degradation, resulting in the failure of gel formation. It follows that a sufficient molecular weight of polysaccharide is critical for the gel properties [51].

### 3.5. Cryo-SEM Microstructure of Carrageenan Degradation Products

**Figure 10:**
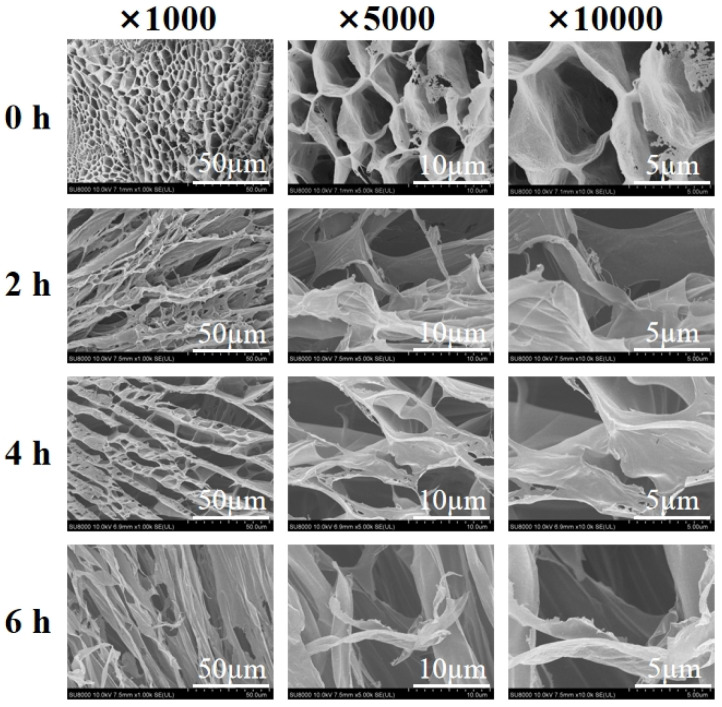
Cryo-SEM images of the samples of κ-carrageenan, DC-2, DC-4, and DC-6. (Magnifications: ×1000, ×5000, and ×10,000).

Cryo-SEM has long been used for direct observation of the retained microstructure of the samples [52]. The microstructure of κ-carrageenan, DC-2, DC-4, and DC-6 were characterized by Cryo-SEM, and the results were shown in Figure 10. The gel pore wall thickness and pore area were determined with ImageJ; κ-carrageenan, DC-2, DC-4, and DC-6 had a pore wall thickness of 0.29 ± 0.06 μm, 0.16 ± 0.01 μm, 0.14 ± 0.02 μm, and 0.12 ± 0.01 μm, respectively. In addition, the pore area of κ-carrageenan was 21.50 ± 4.18 μm^2^ while the pore structures of the degradation products were fragmented.

Overall, κ-carrageenan showed a typical gel network structure, which had large pores and thick and homogeneous pore walls. Notably, the pore walls of degradation products became thinner, and imperfectly linked with some filamentous structures. It could be noticed that with the extension of photocatalytic time, the degradation products became irregular and broken. The microstructure features are in line with the result that the degradation products would lose their gel-like behavior with the increase of degradation time. 

## 4. Conclusions

The present study developed a photocatalytic method for carrageenan degradation and by using TiO_2_ as the only catalyst, this method could decrease the average molecular weight of κ-carrageenan to 4 kDa within 6 h. Notably, no significant loss of sulfate groups in the reaction was observed, which is critical for its application in preparing sulfated degradation products. Moreover, the degradation rules were proposed according to the degradation product characterization, and the glycosidic bond of AnGal was found to be more susceptible to break. In addition, the present study demonstrated the decreasing gel property of carrageenan alone with the photocatalytic degradation. Thus, the present study indicates the promising application of photocatalytic degradation with TiO_2_ to prepare sulfated low-molecular-weight carrageenan, which may further broaden the application of carrageenan.

## Figures and Tables

**Table 1:** ^1^H and ^13^C NMR chemical shifts of DC-6 in D_2_O.

Glycosyl Residues	^13^C/^1^H (ppm)					
	C1/H1	C2/H2	C3/H3	C4/H4	C5/H5	C6/H6
G: Gal4S	102.1/4.63	69.2/3.58	78.1/3.99	73.7/4.83	74.5/3.79	61.2/3.79
A: AnGal	94.5/5.08	69.3/4.12	78.7/4.51	77.8/4.60	76.5/4.63	69.2/4.18

**Table 2:** Relative contents of κ-carrageenan oligosaccharides detected in DC-6.

No.	Retention Time (min)	*m*/*z*	Oligosaccharide Chain	Area Ratio (%)
1	5.68	655	AnGal→Gal	13.50
2	29.27	735	AnGal→Gal→SO_3_	0.45
3	5.53/30.22	799	AnGal→Gal→AnGal	10.54
4	21.63/29	817	Gal→AnGal→Gal	21.05
5	17.31	849	dAnGal→Gal→AnGal→SO_3_	3.74
6	22.45	931	dAnGal→Gal→AnGal→Gal	9.38
7	21.63/29.13	962	AnGal→Gal→AnGal→Gal	19.57
8	22.45/29.62	1011	dAnGal→Gal→AnGal→Gal→SO_3_	1.63
9	21.63/29.13	1041	AnGal→Gal→AnGal→Gal→SO_3_	4.05
10	29.62	1075	dAnGal→Gal→AnGal→Gal→AnGal	2.26
11	40.01	1123	Gal→AnGal→Gal→AnGal→Gal	2.60
12	29.62	1155	dAnGal→Gal→AnGal→Gal→AnGal→SO_3_	0.52
13	29.23	1185	AnGal→Gal→AnGal→Gal→AnGal→SO_3_	1.40
14	39.93	1203	Gal→AnGal→Gal→AnGal→Gal→3SO_3_	0.73
15	40.18	1267	AnGal→Gal→AnGal→Gal→AnGal→Gal	4.28
16	40.18	1347	AnGal→Gal→AnGal→Gal→AnGal→Gal→SO_3_	1.31
17	40.24	1363	Gal→AnGal→Gal→AnGal→Gal→SO_3_	0.64
18	41.3	1411	AnGal→Gal→AnGal→Gal→AnGal→Gal→AnGal	1.52
19	41.3	1491	AnGal→Gal→AnGal→Gal→AnGal→Gal→AnGal→SO_3_	0.46
20	41.3	1507	AnGal→Gal→AnGal→Gal→AnGal→Gal→3SO_3_	0.37

## Data Availability

Not applicable.
